# Examination of Physicians' Perception of the Indications of Colorectal Stents in the Management of Malignant Large Bowel Obstruction: A Provincial Survey

**DOI:** 10.1155/2016/4629710

**Published:** 2016-09-20

**Authors:** Jean-Frédéric LeBlanc, Myriam Martel, Alan N. Barkun

**Affiliations:** ^1^McGill University Health Centre, Montreal, QC, Canada; ^2^Division of Gastroenterology, McGill University Health Centre, Montreal, QC, Canada

## Abstract

*Introduction*. Data are conflicting when assessing indications for colorectal self-expandable metallic stents (SEMS) in managing acute malignant large bowel obstruction (MLO). In November 2014, European and American Societies published guidelines to aid in understanding which patients might benefit from colorectal stenting. Yet, there remain marked disparities in clinical practice.* Methods*. A web-based survey was sent to Gastroenterologists and Surgical Specialists across Quebec to assess physicians' knowledge and adherence to the indications for colonic SEMS placement in the management of MLO using eight clinical scenarios.* Results*. Out of 112 respondents, 74% preferred surgical intervention in young, healthy individuals with MLO. Advanced age and comorbidities motivated 56.3% (95% CI 47.1–65.5%) of participants to opt for SEMS placement. In palliative settings of patients undergoing chemotherapy including bevacizumab, a minority of respondents followed guidelines, 12.5% (95% CI 6.4–18.6%) for young patients and 25.0% for elderly patients (95% CI 17.0–33.0%). The pooled overall adherence to guidelines was 50.4% (95% CI 40.7–59.3%).* Conclusion*. This survey suggests that guidelines recommendations are not being implemented by at least half of specialists involved in the care of patients with MLO. Future studies should attempt to identify possible barriers responsible for this impaired knowledge translation and tailored educational initiatives planned accordingly.

## 1. Introduction

Despite decreasing incidence, colorectal cancer remains the third most prevalent malignancy worldwide in men and second in women. It ranks as fifth in all-cancer mortality [1]. Colorectal cancer can present or evolve to malignant large bowel obstruction (MLO) in 10–29% of cases [2]. Traditionally, physicians have resorted to urgent decompressive surgery, which often consists of a multiple-step procedure. However, this approach is associated with high mortality and frequent adverse events, such as permanent stomas [3].

Thus, physicians have increasingly opted for insertion of self-expandable metallic stents (SEMS). These are reserved for more distal, nonrectal locations of obstruction [4]. SEMS were introduced in the last twenty years in the goal of temporizing an otherwise inflamed colon [5]. Such a procedure would thus allow malignancy staging (in new cases) and additional time to decide whether surgical management is warranted [6, 7]. However, multiple randomized controlled trials and meta-analyses have failed to show a difference in mortality between emergent surgery and colorectal stents and even suggest increased subsequent oncologic morbidity with stent insertion, such as tumour ingrowth or long-term recurrence [6–10]. In light of conflicting data, the European Society of Gastrointestinal Endoscopy (ESGE), spearheaded by expert task forces, commissioned guidelines to examine the proper indications of SEMS in MLO based on the existing literature. The guidelines were then reviewed and edited by experts from the American Society of Gastrointestinal Endoscopy (ASGE) and published in both societies' respective journals [4, 11].

The aim of the study is thus to assess physicians' knowledge and adherence to the indications of colorectal stents in the management of acute MLO.

## 2. Methods

### 2.1. Survey Participants

Targeted responders were staff Gastroenterologists and Surgeons who are currently members of their respective Quebec-based specialty associations. However, we did not have access to a detailed database that could limit the invitation to endoscopists who insert colonic stents. A standardized bilingual email was sent to the secretary of each association, who forwarded it to its respective members with the permission of each society's executive.

### 2.2. Survey Design

The survey was constructed in light of the published recommendations on appropriate conduct of survey research [12]. The content of the survey was separated into four broad categories: baseline demographics, physicians' case exposure, clinical scenarios, and review of guidelines.

The first page described the authors' background, the aims of the study, and the agreement summary. Participants were identified through age, sex, specialty, type of practice, and practice length. Specific data regarding their practice was then collected: types of procedures performed (colonoscopies, colorectal stent insertion) and individual case exposure to MLO and to SEMS as bridge to surgery or in a palliative intent.

Eight clinical scenarios (Box 1) described different case presentations, where key prognostic factors were explicitly identified in keeping with characteristics critical to decision-making as per guidelines. Age and extent of comorbidity were the first studied parameters. Extent of disease and potential for chemotherapy were also assessed. Participants could choose from four therapeutic options for each clinical scenario, as described here: “Option A: Insert a colorectal stent, with view to decompressive surgery in 5–10 days; Option B: Insert a colorectal stent regardless of whether the patient may have subsequent surgery or not; Option C: Send patient to the operating room for urgent decompressive surgery; Option D: Observe patient's symptoms for 24–48 hours with nasogastric suction.”

After completing the clinical scenarios section, the respondents could then access a summarized version of the 2014 ESGE/ASGE guidelines. Multiple-choice questions assessed physicians' awareness of the current guidelines and their perception. In the context of respondents' disagreement, up to five explanations could be selected, including the choice “Other” enabling access to a free text response. Finally, respondents' were asked whether the data presented in this survey would change their practice. English and French versions of the survey were available upon request.

### 2.3. Guidelines Content

A brief summary of the guidelines regarding the indications of SEMS in MLO, established by the European and American Societies of Gastrointestinal Endoscopy, was included in the survey. The text is shown here: “Both the American and European Societies of Gastrointestinal Endoscopy agree that colorectal stents are not recommended in malignant large bowel obstruction as a bridge to surgery, except for patients with an age ≥ 70 or ASA score ≥ III. These international societies also state that colorectal stents are recommended in malignant large bowel obstruction in a palliative intent, except in patients treated with anti-angiogenic chemotherapy (bevacizumab) [4].”

### 2.4. Survey Distribution and Ethical Considerations

The potential participants received an invitation email on October 22, 2015, referring to the online survey supported by* SurveyMonkey* (https://www.surveymonkey.com/). The first answer was recorded on October 22, 2015, and the last on November 11, 2015. The survey was available in French and English. Participation was voluntary. Respondents were allowed to stop the survey at any time. They were asked to agree to participate and provided informed consent prior to initiating the survey. The study was approved by the McGill University Health Centre, Research Ethics Board.

### 2.5. Analytical Plan

Descriptive statistics including means, medians, and standard deviations for continuous variables, as well as proportions with 95% confidence intervals (95% CI) for categorical variables, were used for the main results. Categorical data were also analyzed with *χ* ^2^-test or Fisher's exact test. *P* values of less than 0.05 were considered statistically significant.

It was decided* a priori* that results of incompletely filled surveys would be excluded to avoid potential overlapping responses because of the way the* SurveyMonkey* tool processes collected data.

Statistical analyses were performed with the program SAS version 9.3 (SAS Institute, Cary, NC, USA).

## 3. Results

### 3.1. Baseline Characteristics

The survey was sent to 500 Surgeons and 237 Gastroenterologists and Hepatologists through their respective Quebec associations. In total, 124 (16.8%) participants responded to the survey, including 12 who did not completely fill out the survey. Their answers were thus excluded from the present study.

The overall response rate was 15.2% (112/737). As seen in Table 1, 92.0% of participants completed the survey in French. All of the respondents were staff physicians. They were either Gastroenterologists (39.3%) or Surgeons of the following specialties (58.9%): General Surgery (61), Colorectal Surgery (4), and Surgical Oncology (1). One respondent, from General Internal Medicine, was included in the study in the context of his current endoscopic practice as he was identified by the Gastroenterologists' database. A total of 86.6% of respondents performed endoscopies. Overall, 19.6% of physicians inserted colorectal stents as part of their practice and, of these respondents, 81.8% were staff Gastroenterologists.

Type of practice was almost equally divided between academic (42%) and community (40.2%) settings. Otherwise, 17.8% of respondents practiced in both settings. Complete baseline participant characteristics are listed in Table 1.

### 3.2. Physician Experience and Continued Education Sources

In the twelve months prior to the survey, 39.2% (44/112) and 9.8% (11/112) of respondents had been consulted on 5–10 and more than 10 cases of MLO, respectively. Overall, 51% (57/112) were involved in less than five cases of MLO.

**Figure 1:**
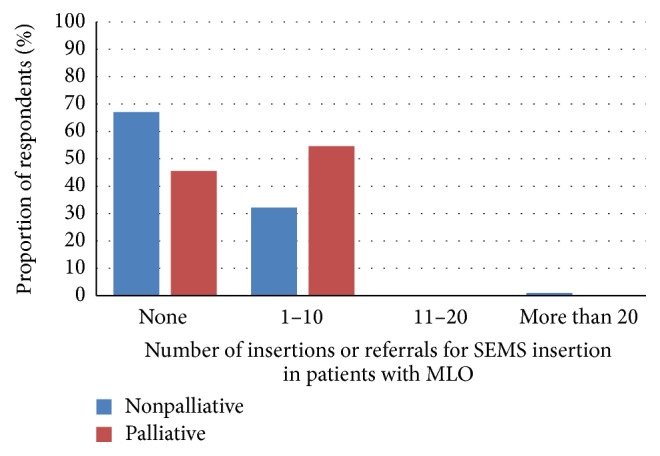
This figure shows the respondents' utilization of self-expandable metallic stents (SEMS) in the management of malignant large bowel obstruction (MLO) in palliative and nonpalliative settings in the twelve months prior to the survey.

In the same time period, approximately 32% of respondents treated 1–10 cases of MLO by inserting SEMS or by referring for insertion as a bridge to surgery; 54.4% of respondents did so when treating a patient in a palliative intent (see Figure 1). Only one respondent used colorectal stents in a nonpalliative intent as his main therapeutic strategy in more than 20 cases of MLO.

Most respondents (75%) used medical conferences as a means to update their knowledge for the indications of SEMS in the management of acute MLO. Primary journal articles and online clinical resources were consulted by approximately half of respondents (51.2%). Only 20.5% of participants relied on clinical guidelines for knowledge of SEMS indications.

### 3.3. Clinical Scenarios

**Figure 2:**
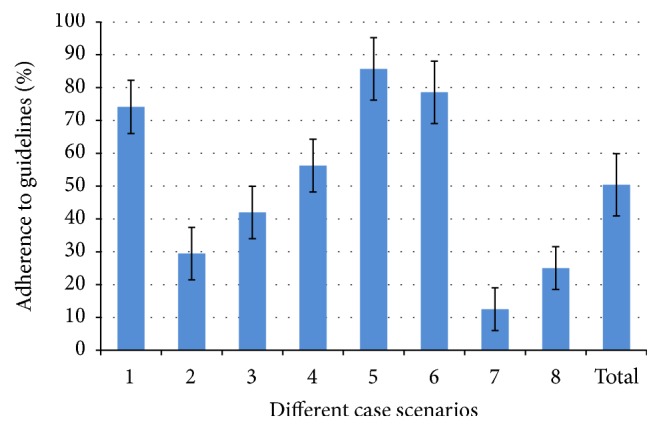
This figure shows the adherence of respondents to recently published guidelines (European and American Societies of Gastrointestinal Endoscopy, 2014), assessing the indications of colorectal stents in the management of acute malignant large bowel obstruction. Eight clinical scenarios were formulated as shown in Box 1. Participants could choose from four therapeutic options (as described below), one of which was deemed to be in keeping with guidelines. A pooled analysis was performed taking into account all case scenarios, yielding an overall rate of adherence of 50.4% (95% CI 40.7–59.3%) of participants opting for a therapeutic strategy recommended by guidelines. The therapeutic options are clarified: “Option A: Insert a colorectal stent, with view to decompressive surgery in 5–10 days; Option B: Insert a colorectal stent regardless of whether the patient may have subsequent surgery or not; Option C: Send patient to the operating room for urgent decompressive surgery; Option D: Observe patient's symptoms for 24–48 hours with nasogastric suction."

Respondents' therapeutic decisions based on eight clinical scenarios are summarized in Table 2. The participants' adherence to recently published guidelines regarding SEMS insertion for cases of MLO is shown in Figure 2.

In the first case scenario (60-year-old otherwise healthy), 74.1% (95% confidence interval (CI) 66.0–82.2%) of respondents opted for a surgical approach, in keeping with guidelines.

In the second (elderly otherwise healthy) and third (60-year-old with comorbidities) scenarios, a minority of participants followed guidelines: 29.5% (95% CI 21.1–38.0%) and 42.0% (95% CI 32.9–51.1%), respectively.

With regard to the fourth case scenario (elderly patient, with comorbidities), a majority of respondents opted for SEMS insertion, as recommended by the guidelines: 56.3% (95% CI 47.1–65.5%) [4].

For palliative scenarios 5 and 6 that represented patients not undergoing bevacizumab chemotherapy, respondents agreed with guidelines: 85.7% (95% CI 79.2–92.2%) and 78.6% (95% CI 71.0–86.2%), respectively.

For patients receiving bevacizumab chemotherapy (cases 7 and 8), a significant minority opted for conservative management, in keeping with ESGE preferred management: 12.5% (95% CI 6.4–18.6%) and 25.0% (95% CI 17.0–33.0%), respectively.

A pooled analysis was performed taking into account all case scenarios, yielding an overall adherence rate of 50.4% (95% CI 40.7–59.3%) of participants opting for a therapeutic strategy recommended by guidelines.

As a subgroup analysis, results were restricted to respondents having been exposed to 5 cases or more of acute MLO in the previous twelve months (as seen in Table 3). The pooled adherence rate was 48.0% (95% CI 34.8–61.2%), compared to 53.3% (95% CI 40.4–66.3%) for the respondents exposed to less than 5 cases of MLO in the same time period (*P* = 0.71).

In a second subgroup analysis, results were compiled strictly from physicians who insert colorectal stents as part of their practice (Table 4). Pooled adherence to guidelines was calculated at 51.1% (95% CI 41.8–60.4%), compared to 50.3% (95% CI 40.0–60.6%) for respondents who do not insert SEMS (*P* = 0.98).

### 3.4. Awareness of Current Guidelines

Out of the 112 respondents, 42 (37.5%) were aware of the 2014 guidelines from the European and American Societies of Gastrointestinal Endoscopy. Moreover, 63.6% (95% CI 43.5–83.7%) of respondents who insert SEMS were cognizant of the guidelines, compared to 30.0% (95% CI 20.5–39.5%) of physicians who do not insert SEMS, *P* = 0.0058. The articles, published in* Gastrointestinal Endoscopy *or* Endoscopy*, and their abstracts were read, respectively, by 7.1% and 13.4% of participants.

A total of 65.2% slightly agreed with these recommendations, while 12.5% strongly agreed. Of the 22 endoscopists who insert colorectal stents, 68.2% strongly or slightly agreed with the guidelines.

Reasons for disagreement differed. Participants estimated patients should be managed on a case-by-case basis (33%) or that there was lack of high-quality evidence (14.3%). Certain respondents (13.4%) considered there should not be an exception for cases treated as bridge to surgery (either age ≥ 70 or American Society of Anaesthesia (ASA) score of ≥ III). One respondent mentioned that, in his practice, colonic SEMS are inserted in prophylactic measures without clinically evident intestinal obstruction.

Overall, 43.8% did not believe that the guidelines would change their practice, while 38.4% suggested their practice may change in acknowledgment of these recommendations.

## 4. Discussion

Management of acute MLO has become more controversial in the last decade, especially in cases of distal nonrectal colonic obstruction [13]. Multiple RCTs and meta-analyses have examined the potential benefit of colonic SEMS. Colonic stents avoid the need for a multistage surgery and provide improved short-term outcomes, such as higher rates of primary anastomosis, lower rates of permanent stomas, and improved quality of life [6, 7].

However surprisingly, these do not result in subsequent mortality benefit [14]. In fact, small-sized RCTs have suggested a potential harm related to the insertion of SEMS for nonpalliative disease in terms of long-term oncologic risk with increased recurrences [8, 9]. In addition, there appears to be an increased risk of stent-induced perforations in patients receiving bevacizumab [10].

The disparity between short- and long-term outcomes is counterintuitive and may explain why a full 50.4% (95% CI 40.7–59.3%) of surveyed physicians across all case scenarios did not adhere to existing guidelines in their choice of management strategies.

Survey responses showed that physicians prefer a surgical approach in managing young, healthy individuals when the intent is considered nonpalliative, in keeping with guidelines. The degree of comorbidities seemed to be considered a poor prognostic factor, motivating 42% of participants to opt for SEMS placement in a young, comorbid patient instead of a surgical approach, compared to 29.5% of respondents assessing an elderly yet healthy patient. A single risk factor did not translate into increased SEMS insertion preference, contrary to guidelines; however, advanced age and comorbidities motivated a majority (56.3%, 95% CI 47.1–65.5%) of respondents to opt for SEMS insertion, as per guidelines [4]. Published data suggest that age and comorbidities are the predominant predictors of in-hospital mortality in acute MLO [15].

In a palliative setting where chemotherapy was not part of the therapeutic regimen, a majority of respondents opted for insertion of colorectal stents regardless of whether surgery might be subsequently contemplated. These management choices are in keeping with guidelines. Benefit has been shown in large meta-analyses, which have included RCTs, and prospective and retrospective cohort studies. In the recent large meta-analysis by Zhao et al., 837 palliative cases of acute MLO were assessed. Results suggested a lower mortality risk in SEMS-treated patients in this population versus emergency surgery (4* *% versus 11* *%, resp.). SEMS insertion was also associated with shorter hospitalization, shorter time to initiation of chemotherapy, and lower stoma formation [16].

In the survey, most respondents opted for SEMS placement when a chemotherapy regimen including bevacizumab was part of the overall treatment strategy. Guidelines, however, have recommended against SEMS insertion in patients receiving antiangiogenic therapy (strong recommendation, low quality of evidence), such as bevacizumab, a vascular-endothelial-growth-factor (VEGF) inhibitor [4]. Indeed, a meta-analysis has confirmed a higher risk of SEMS-associated perforations in such patients (12.5%) compared to chemotherapy protocols without a VEGF inhibitor (7.0%) [10]. The optimal approach in palliative patients treated with VEGF inhibitors has yet to be defined.

A first subgroup analysis was conceived in the goal of assessing a possible association between increased exposure to MLO, defined as 5 cases or more of MLO in the previous twelve months, and adherence to guidelines. The pooled adherence rate was 48.0% (95% CI 34.8–61.2%), compared to 53.3% (95% CI 40.4–66.3%) for the respondents exposed to less than 5 cases of MLO yearly (*P* = 0.71), suggesting that increased exposure to MLO did not translate to better adherence to guidelines, although not statistically significant in this study.

A second subgroup analysis examined the hypothesis of a higher adherence to guidelines in respondents who insert SEMS as part of their practice. Of note, these clinicians seemed to have a higher awareness of the guidelines: of all the respondents who insert SEMS, 63.6% (95% CI 43.5–83.7%) were aware of the guidelines, compared to 30.0% of physicians who do not insert SEMS (95% CI 20.5–39.5%), *P* = 0.0058.

Yet, it did not translate to improved adherence rates, perhaps because only 68.2% (95% CI 48.7–87.7%) of respondents who insert SEMS strongly or slightly agreed with the recommendations; however, this proportion was not statistically different from the 80.0% (95% CI 71.7–88.3%) agreement amongst those who do not insert SEMS, *P* = 0.26.

Interestingly, no difference in pooled adherence to guidelines was noted between both groups: 51.1% (95% CI 30.2–72.0%) for respondents who insert colorectal stents, compared to 50.3% (95% CI 40.0–60.6%) for those who do not insert SEMS, *P* = 0.98. This observation may be attributable to individual situations made on a case-by-case basis, in which difficult clinical situations offer little in terms of therapeutic alternatives other than stent insertion.

The statistical significance of our results was limited by a small sample size of physicians who insert SEMS (19.6% of overall respondents).

### 4.1. Limitations of the Study

The response rate was low in this study (15.2%); however this may be explained by the realization that, out of the 737 Quebec Gastroenterologists and Surgeons, we believe that only few insert colorectal stents in their practice probably less than 20% based on local experience (Barkun A, personal communication) and others may not have felt the need to respond in any way to the survey. Taking this participation and the descriptive demographics into consideration, we thus believe that the results may be generalizable to the level of province-wide practice and beliefs, other than that of response bias with the recognized overestimation of evidence-based knowledge by a professional choosing to respond to the invitation to participate in the survey in the first place [17]. Respondents were allowed to backtrack during the survey. Potentially, therapeutic decisions to the eight scenarios could have been modified after the respondents were aware of the guidelines.

It is thus important to note that the upper margin of the pooled adherence rate suggests that at best 59.3% of Quebec physicians adhere to existing MLO management guidelines. In retrospect, the opinion of Interventional Radiologists would also have been interesting to assess as some SEMS are now being inserted fluoroscopically [18], even though only a small subgroup performs endoscopic stent insertion in Quebec.

Other limitations include use of the ASA score as measure of perioperative risk prediction in the published guidelines [4, 11]. A score of III or more refers to “severe systemic disease,” a descriptive term that might have been perceived as subjective by respondents of the survey. A systematic review suggests that opting for numerical scoring systems may lead to better triage and management of patients requiring emergent laparotomy, such as the Acute Physiology and Chronic Health Evaluation II (APACHE II) score or the Physiologic and operative severity score for the enumeration of morbidity and mortality (POSSUM) score [19].

Respondents' suggested approaches seemed speculative in certain settings despite high-quality evidence. This could be explained by poor translation of best current evidence to clinical practice, otherwise known as knowledge translation (KT) or implementation. The Canadian Institutes of Health Research (CIHR) describes KT as “a dynamic and iterative process that includes the synthesis, dissemination, exchange and ethically-sound application of knowledge to improve health services and products and strengthen the healthcare system” [20].

A crucial part of KT is identifying and measuring the gap between evidence and practice. In order to apply KT projects, barriers to a behavioural change need to be examined [21]. In the current study, we believe that the barriers are multileveled, from the individual physician to the complex health care organization, although a formal needs-barrier analysis was not carried out because of the aims and scope of the study. Respondents might lack awareness of current guidelines; they might also disagree with the existing evidence to justify a therapeutic option for their individual patient. Discordance might also stem from external factors such as insufficient resources to put knowledge into practice, as well as inadequate support from health care organizations.

This survey allows assessment of the management choices by Gastroenterologists and Surgical Specialists in the treatment of MLO. The perception is that current evidence suggests that SEMS are an acceptable therapeutic option in selected cases of MLO. Unfortunately, it would appear that recommendations from recently published guidelines are neither poorly known, nor thoroughly applied to practice. Indeed only 37.5% (42/112) of respondents had read the guidelines publications or their abstracts. The pooled overall adherence to guidelines across all proposed scenarios in the survey was 50.4% (95% CI 40.7–59.3%), suggesting a significant gap between evidence and practice. Further studies, appropriate needs-barrier analyses, improved knowledge dissemination, and more formal implementation of an educational strategy should be contemplated by professional societies and regulating bodies to address optimal management of MLO.

## Figures and Tables

**Box 1:**
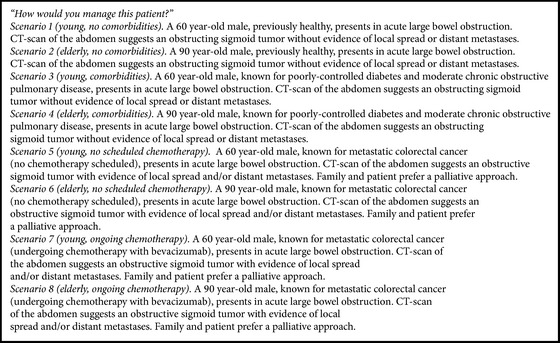
Clinical scenarios of different cases of malignant large bowel obstruction.

**Table 1:** Baseline characteristics of survey respondents.

Demographics (*n* = 112)		
Survey language	English	8.0% (9)
	French	92.0% (103)

Age	Less than 35	25.9% (29)
	36–45	33.9% (38)
	46–55	25.0% (28)
	56–65	12.5% (14)
	More than 65	2.7% (3)

Sex	Male	64.3% (72)
	Female	35.7% (40)

Specialty	Gastroenterology	39.3% (44)
	Surgical	58.9% (66)
	Other	1.8% (2)

Type of practice	Academic	42.0% (47)
	Community	40.2% (45)
	Both	17.8% (20)

Years of practice	Less than 10	39.3% (44)
	10–19	33.0% (37)
	20 or more	27.7% (31)

Colonoscopies	Yes	86.6% (97)
	No	13.4% (15)

Colorectal stents	Yes	19.6% (22)
	No	80.4% (90)

**Table 2:** All respondents' management decisions (measured in proportions, %) based on eight clinical scenarios of malignant large bowel obstruction (detailed in Box 1), with the optimal approach highlighted in bold, as suggested by the 2014 ESGE/ASGE guidelines.

Scenario	Option A	Option B	Option C	Option D
1: young, healthy	17.9	0	**74.1**	8
2: elderly, healthy	**29.5**	20.5	39.3	10.7
3: young, comorbid	**42**	7.1	42.9	8
4: elderly, comorbid	22.3	**56.3**	13.4	8
5: young, no chemotherapy	4.5	**85.7**	6.2	3.6
6: elderly, no chemotherapy	0.9	**78.5**	2.7	17.9
7: young, chemotherapy	9.8	57.1	20.6	**12.5**
8: elderly, chemotherapy	0.9	64.3	9.8	**25**

The therapeutic options are clarified: “Option A: Insert a colorectal stent, with view to decompressive surgery in 5–10 days; Option B: Insert a colorectal stent regardless of whether the patient may have subsequent surgery or not; Option C: Send patient to the operating room for urgent decompressive surgery; Option D: Observe patient's symptoms for 24–48 hours with nasogastric suction.”

**Table 3:** A subgroup of respondents' management decisions (measured in proportions, %) based on eight clinical scenarios of malignant large bowel obstruction or MLO (detailed in Box 1), with the optimal approach highlighted in bold, as suggested by the 2014 ESGE/ASGE guidelines. Only the therapeutic choices of respondents who were exposed to at least five cases of MLO in the previous twelve months are shown here.

Scenario	Option A	Option B	Option C	Option D
1: young, healthy	16.4	0	**74.5**	9.1
2: elderly, healthy	**21.8**	21.8	45.5	10.9
3: young, comorbid	**36.4**	10.9	41.8	10.9
4: elderly, comorbid	27.3	**47.3**	16.3	9.1
5: young, no chemotherapy	5.4	**85.6**	3.6	5.4
6: elderly, no chemotherapy	0	**74.6**	3.6	21.8
7: young, chemotherapy	9.1	61.9	14.5	**14.5**
8: elderly, chemotherapy	0	65.5	5.4	**29.1**

The therapeutic options are clarified: “Option A: Insert a colorectal stent, with view to decompressive surgery in 5–10 days; Option B: Insert a colorectal stent regardless of whether the patient may have subsequent surgery or not; Option C: Send patient to the operating room for urgent decompressive surgery; Option D: Observe patient's symptoms for 24–48 hours with nasogastric suction.”

**Table 4:** A subgroup of respondents' management decisions (measured in proportions, %) based on eight clinical scenarios of malignant large bowel obstruction or MLO (detailed in Box 1), with the optimal approach highlighted in bold, as suggested by the 2014 ESGE/ASGE guidelines. Only the therapeutic options of respondents who insert colorectal stents as part of their practice are shown here.

Scenario	Option A	Option B	Option C	Option D
1: young, healthy	27.3	0	**59.1**	13.6
2: elderly, healthy	**40.8**	22.8	27.3	9.1
3: young, comorbid	**54.6**	4.5	27.3	13.6
4: elderly, comorbid	27.3	**50**	9.1	13.6
5: young, no chemotherapy	4.5	**91**	0	4.5
6: elderly, no chemotherapy	0	**77.3**	0	22.7
7: young, chemotherapy	13.6	50	22.8	**13.6**
8: elderly, chemotherapy	0	72.7	4.5	**22.8**

The therapeutic options are clarified: “Option A: Insert a colorectal stent, with view to decompressive surgery in 5–10 days; Option B: Insert a colorectal stent regardless of whether the patient may have subsequent surgery or not; Option C: Send patient to the operating room for urgent decompressive surgery; Option D: Observe patient's symptoms for 24–48 hours with nasogastric suction.”
